# AI Dermatochroma Analytica (AIDA): Smart Technology for Robust Skin Color Classification and Segmentation

**DOI:** 10.3390/cosmetics11060218

**Published:** 2024-12-10

**Authors:** Abderrachid Hamrani, Daniela Leizaola, Nikhil Kumar Reddy Vedere, Robert S. Kirsner, Kacie Kaile, Alexander Lee Trinidad, Anuradha Godavarty

**Affiliations:** 1 Department of Mechanical and Materials Engineering, Florida International University, Miami, FL 33174, USA; 2 Optical Imaging Laboratory, Department of Biomedical Engineering, Florida International University, 10555 West Flagler Street, EC 2675, Miami, FL 33174, USA; 3 Department of Dermatology & Cutaneous Surgery, University of Miami Miller School of Medicine, Miami, FL 33174, USA

**Keywords:** skin color classification, machine learning unsupervised clustering

## Abstract

Traditional methods for skin color classification, such as visual assessments and conventional image classification, face limitations in accuracy and consistency under varying conditions. To address this, we developed AI Dermatochroma Analytica (AIDA), an unsupervised learning system designed to enhance dermatological diagnostics. AIDA applies clustering techniques to classify skin tones without relying on labeled data, evaluating over twelve models, including K-means, density-based, hierarchical, and fuzzy logic algorithms. The model’s key feature is its ability to mimic the process clinicians traditionally perform by visually matching the skin with the Fitzpatrick Skin Type (FST) palette scale but with enhanced precision and accuracy using Euclidean distance-based clustering techniques. AIDA demonstrated superior performance, achieving a 97% accuracy rate compared to 87% for a supervised convolutional neural network (CNN). The system also segments skin images into clusters based on color similarity, providing detailed spatial mapping aligned with dermatological standards. This segmentation reduces the uncertainty related to lighting conditions and other environmental factors, enhancing precision and consistency in skin color classification. This approach offers significant improvements in personalized dermatological care by reducing reliance on labeled data, improving diagnostic accuracy, and paving the way for future applications in diverse dermatological and cosmetic contexts.

## 1. Introduction

Dermatological research today faces a significant challenge in the accurate classification and analysis of skin colors [1]. The vast diversity and complexity of human skin colors call for advanced methods capable of discerning subtle variances. Traditional approaches in skin color classification, predominantly relying on subjective visual assessments [2], address the skin’s reaction to light exposure rather than its actual color, highlighting a critical limitation in comprehensively representing the true spectrum of skin colors [3].

In an effort to advance beyond these traditional methods, the field has seen the adoption of conventional imaging technologies and digital photography. However, these technologies often inadequately represent the full range of skin colors, especially under variable lighting conditions [4]. Moreover, the emergence of artificial intelligence (AI) systems utilizing convolutional neural networks (CNNs) [5,6] offers a more objective stance but is hindered by their dependence on extensive labeled datasets, and these systems struggle to address the inherent diversity and complexity of skin colors [7–12].

To tackle these challenges, this paper introduces AI Dermatochroma Analytica (AIDA), an artificial intelligence (AI) framework designed to develop the classification of skin color analysis within the field of dermatology. This system mimics the visual process that clinicians traditionally perform by matching the dominant skin color to the Fitzpatrick Skin Type (FST) scale but enhances precision and objectivity through clustering algorithms that eliminate subjectivity and ensure consistent results. Built upon the concept of employing AI for the alignment of segmented skin image clusters with the Fitzpatrick color scale clusters, AIDA surpasses the constraints of conventional methods.

Central to our methodological framework is the Fitzpatrick skin type scale (FST) [13–15], an established dermatological standard for skin color classification. Incorporating this scale into AIDA ensures alignment with dermatological benchmarks while providing a solid basis for the system’s performance evaluation. Fundamentally, the flexibility of our approach allows for the adaptation to various skin color scales, broadening the scope of its application in diverse dermatological contexts. This study aims to showcase the enhanced efficacy of AIDA over conventional supervised learning models like convolutional neural networks (CNN), which often reintroduce subjectivity and are labor-intensive due to the need for manual labeling. By leveraging unsupervised learning, we aim to capitalize on the ability of these algorithms to unravel complex, non-linear patterns in diverse skin color data, thus overcoming the limitations of current methods that fail to encompass the entire spectrum of human skin colors.

### Background and Related Work

Skin color classification in dermatology has traversed various methodologies, each contributing uniquely to our understanding of and approach towards this complex task. Historically, the classification of skin colors largely depended on subjective visual assessment by clinicians [16]. This method, while being straightforward, suffered from inherent biases and inconsistencies due to individual perception differences [14,17]. The introduction of the Fitzpatrick Skin Type classification system marked a significant step forward. Developed in 1975 by Thomas Fitzpatrick, this scale categorizes skin types based on their response to ultraviolet (UV) light, primarily focusing on the tendency to burn or tan [13–15].

With technological advancements, digital imaging and photography started playing a pivotal role in skin color analysis [18,19]. These methods provided a more objective dataset compared to manual visual assessments. Nevertheless, their effectiveness was often influenced by variability in camera features and settings, such as exposure and white balance. This variability could significantly impact the perception of skin color. Additionally, these techniques fell short in fully capturing the diversity of skin colors across different environmental conditions, highlighting a gap in accurately representing the full spectrum of skin tones [4].

The advent of computerized systems for skin color classification brought a new dimension to this field. These systems, using techniques like colorimetry and spectrophotometry [10], provided more precise and consistent measurements of skin color. They quantified skin color in standardized color spaces such as CIELAB, offering a more reliable approach than subjective visual assessments [19]. However, these methods were still limited by the equipment’s sensitivity and the need for controlled environmental conditions [18,20].

The integration of machine learning (ML), particularly supervised learning models such as CNNs, marked a significant advancement in skin color classification [10,12]. These models brought the promise of learning from large datasets of skin images, offering a more objective and comprehensive analysis. However, their reliance on extensive labeled datasets was a major drawback [11,21,22]. The process of labeling, often requiring expert dermatologists’ input, was time-consuming and potentially reintroduced subjective biases.

While traditional skin color classification methods have provided valuable insights and advancements, they each come with limitations, ranging from subjective biases to technological and practical constraints. The evolution of these methods sets the stage for the development of AIDA, which aims to harness the power of unsupervised machine learning to overcome these challenges and offer a more accurate, efficient, and inclusive approach to skin color classification.

## 2. Materials and Methods

### 2.1. Overview of AIDA System

**Figure 1.**
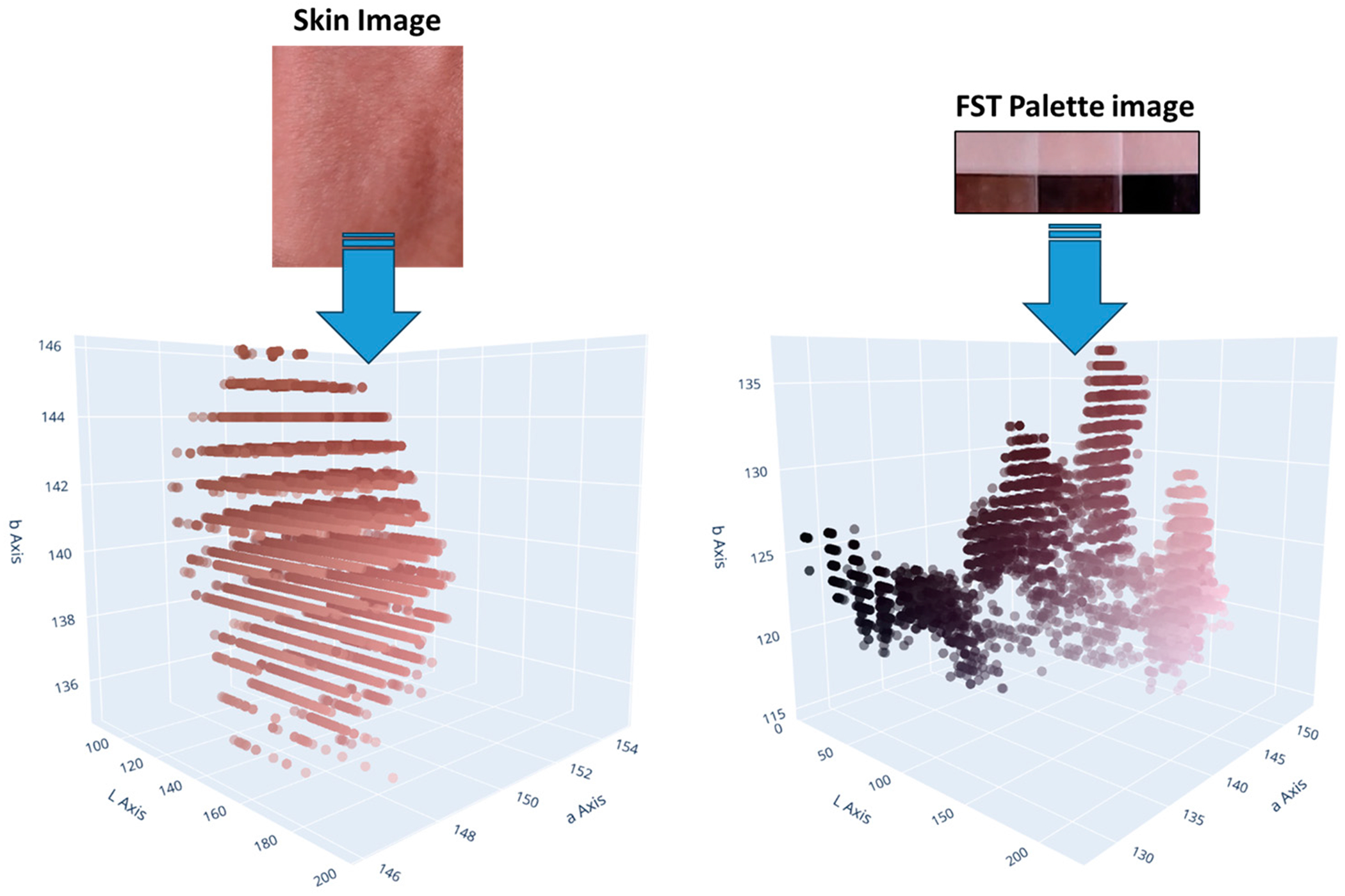
Image transformation to the LAB color space.

**Figure 2.**
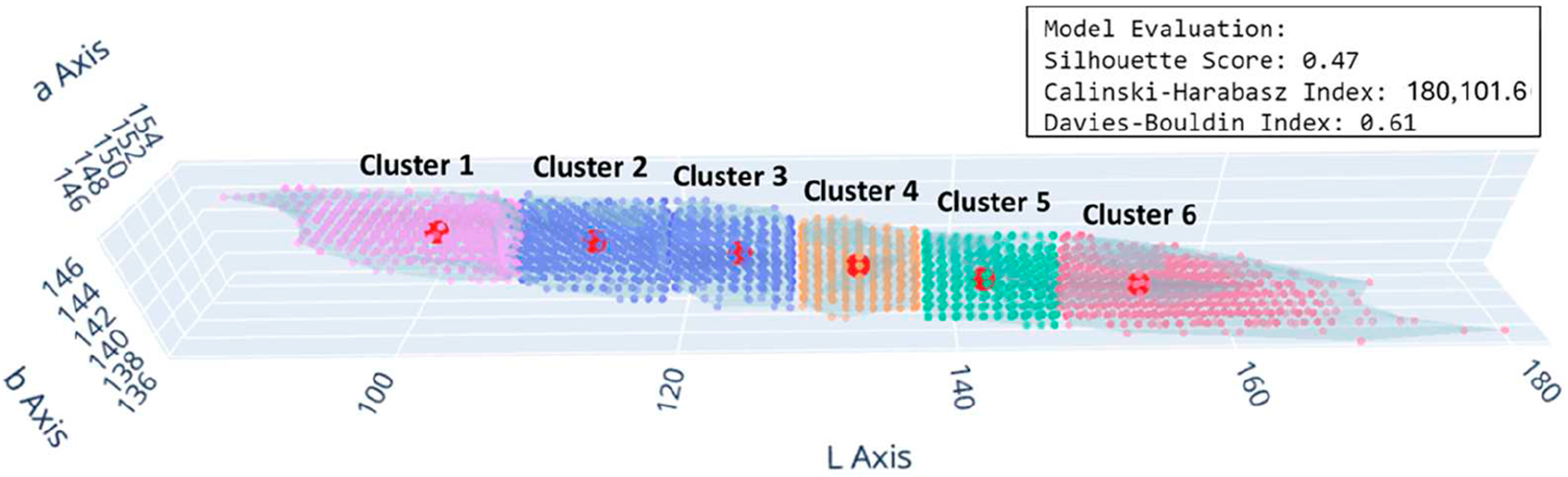
Systematic evaluation and visualization of cluster configurations.

**Figure 3.**
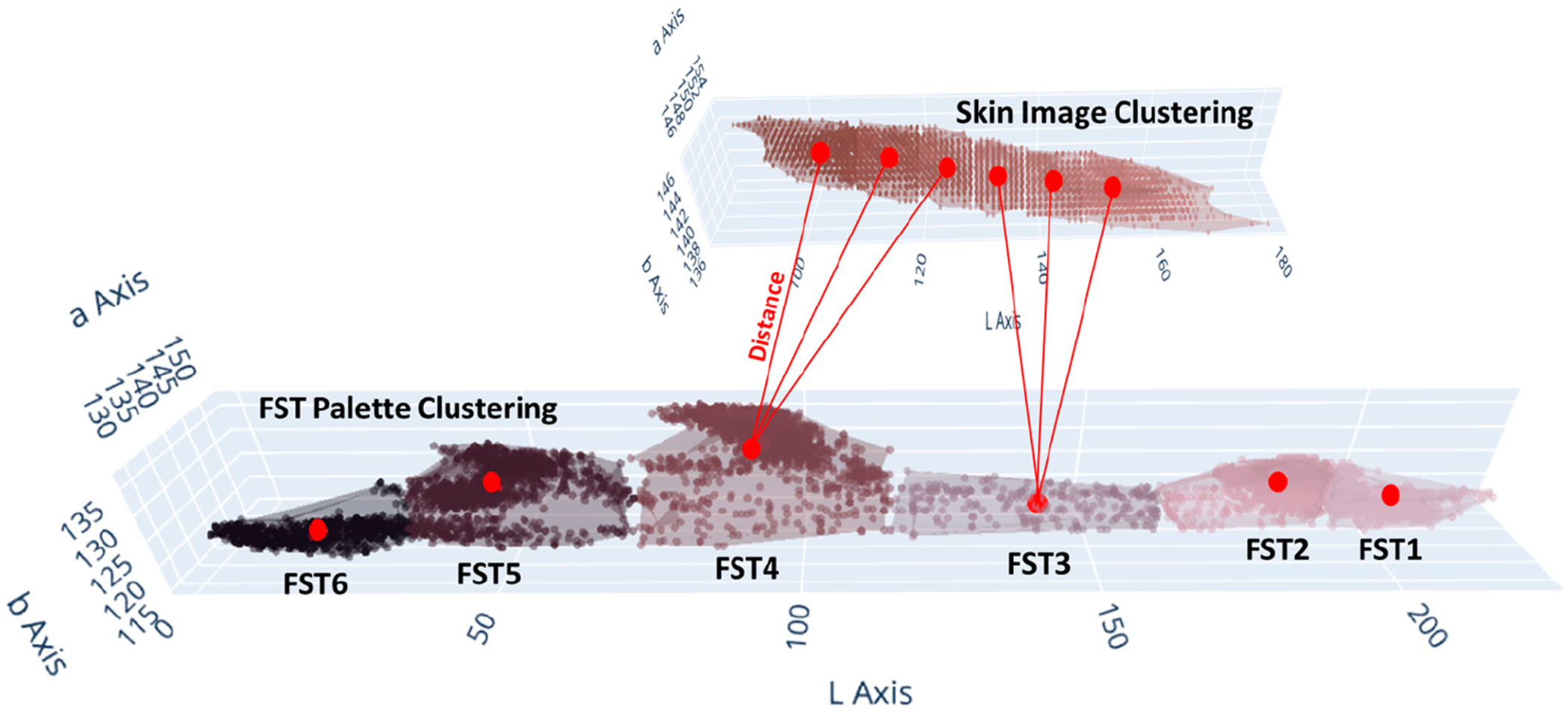
Illustrative scheme of the color matching methodology.

**Figure 4.**
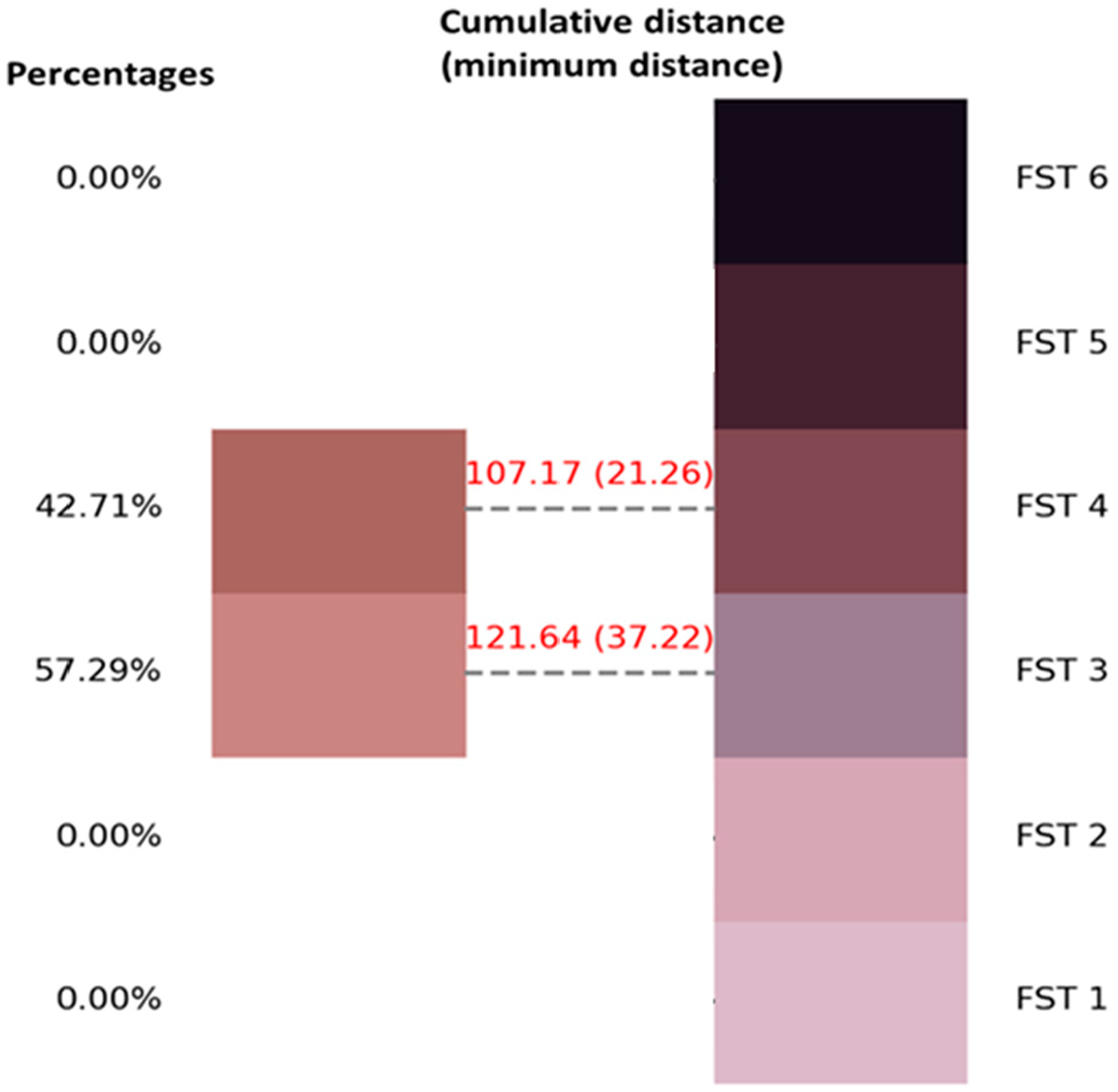
Color alignment visualization between segmented skin colors and Fitzpatrick Skin Type palette.

At the core of AIDA is an unsupervised learning algorithm designed to mimic the process clinicians traditionally perform by visually matching skin tones with the FST palette scale. By leveraging Euclidean distance-based clustering techniques, AIDA enhances precision and accuracy, effectively analyzing complex skin color data. The algorithm used for the AIDA system is as follows:

- Start: Initiate the AIDA system process.
- Import libraries: In the development of the AIDA system, a crucial step involved the importation of various libraries essential for machine learning, image processing, and data visualization. The specific libraries imported and their primary uses in the context of this project are outlined in Appendix A (Table A1).
- Load and preprocess data: The initial phase of loading and preprocessing skin color and FST palette data [23] was essential for the success of subsequent machine learning tasks. This process involved importing the image data and converting these into a more analytically suitable format. The images were transformed from their original color space (R-G-B) to the LAB color space (Figure 1), which is particularly beneficial for skin color analysis due to its ability to provide a nuanced representation of color variations.
- Configure, train, and evaluate clustering model: A methodical approach was adopted for configuring, training, and evaluating the clustering model for image segmentation in the LAB color space. Initially, the parameters of the clustering model, including the number of clusters and the initialization method, were accurately configured. Subsequently, the clustering algorithm was applied to the prepared data. This involved resizing the LAB color space images, reshaping them for the clustering process, and iteratively applying the clustering algorithm until the clusters were optimally formed. The resulting labels and cluster centers were then calculated to provide a detailed segmentation of the image (Figure 2). The quality of clustering was rigorously evaluated using established metrics such as the silhouette score, Calinski–Harabasz Index, and Davies–Bouldin Index. These metrics provided quantitative assessments of the clustering quality, evaluating aspects such as cluster cohesion, separation, and compactness.
- Match cluster centers with FST palette: An essential phase involved the alignment of cluster centers from segmented skin images with the cluster centers of the FST color palette. This key process aimed to determine the closest correspondences between the identified cluster centers of skin colors and those of the FST palette (Figure 3). The first step involved quantifying the perceptual differences between each color in the skin palette (represented by cluster centers) and the colors in the FST palette. This was achieved by calculating the color distance using a standard metric in colorimetry (CIE76 Delta-E color distance), which effectively measures the differences between two colors, i.e., cluster centers. Subsequently, each cluster center from the skin palette was matched with the nearest cluster center in the FST palette based on the calculated color distances. This matching process was fundamental in identifying the most similar FST color for each identified skin color.
- Visualize results: The visualization of results, specifically the alignment of cluster centers with the FST palette, was executed with a specific approach. This process entailed creating visual representations that illustrated the relationship between the segmented skin colors and the FST color palette. The visualization (Figure 4) was designed to display each color from the skin palette alongside its closest match in the FST palette. To enhance the interpretability of these results, the visualizations included the paired colors and annotations indicating the percentage of each skin color within the image and the distance metrics, which quantified the similarity between the skin and FST colors.
- Validation: FST ground-truth classification was determined by corelating ITA measurements from a colorimetry-based tool (Delfin Skin ColorCatch) to the FST skin color scale [24]. This tool was utilized for the validation of the clustering results against real-world skin color measurements.
- End: Conclude the process with validated and calibrated clustering results ready for practical application or further analysis.

### 2.2. Unsupervised Clustering Models

Unsupervised learning, in contrast to its supervised counterpart, does not rely on pre-labeled data, making it uniquely suited for discovering hidden patterns in complex datasets such as those encountered in skin color analysis. Within this framework, diverse arrays of clustering models have been employed and compared to evaluate their strengths and limitations in the task of skin color classification. The clustering models used in AIDA for skin color classification are summarized in Table 1.

By assessing the strengths and limitations of each clustering model in the context of skin color classification, we aim to pinpoint the most effective and accurate method for dermatological analysis.

## 3. Results

In the following sections, the results of the methodologies undertaken in this study are presented. The data collection and preparation steps are first outlined, followed by an examination of the performance metrics employed to evaluate the AIDA system. A comparative analysis of different unsupervised learning models within the AIDA framework is then conducted, leading to a key comparison with a supervised learning model, the convolutional neural network (CNN). Additionally, the segmentation ability of AIDA is examined, highlighting its capacity to partition skin images into distinct clusters that align with dermatological standards.

### 3.1. Data Collection and Preparation

The study design and data collection methodology are presented in Table 2, providing an overview of the steps taken to prepare the data for skin color classification.

### 3.2. Performance Metrics

In the evaluation of the AIDA clustering system, the incorporation of various performance metrics was essential for a rigorous assessment of the clustering models. The performance metrics utilized in this study are enumerated in Table 3.

### 3.3. Comparative Analysis of Clustering Models

In the comparative analysis of different unsupervised learning models conducted within the AIDA framework, a systematic evaluation was undertaken using the performance metrics stated previously (i.e., silhouette score, C-H Index, D-B Index, and training time). The models under consideration included standard K-means, K-means mini-batch, K-means-PCA, DBSCAN, HDBSCAN, OPTICS-DBSCAN, agglomerative hierarchical clustering (AHC), Gaussian mixture models (GMM), fuzzy C-means, affinity propagation, mean shift, and spectral clustering. To ensure a robust and fair evaluation, all clustering models were fine-tuned through hyperparameter optimization, enabling the best possible performance for each model throughout the comparison.

**Figure 5.**
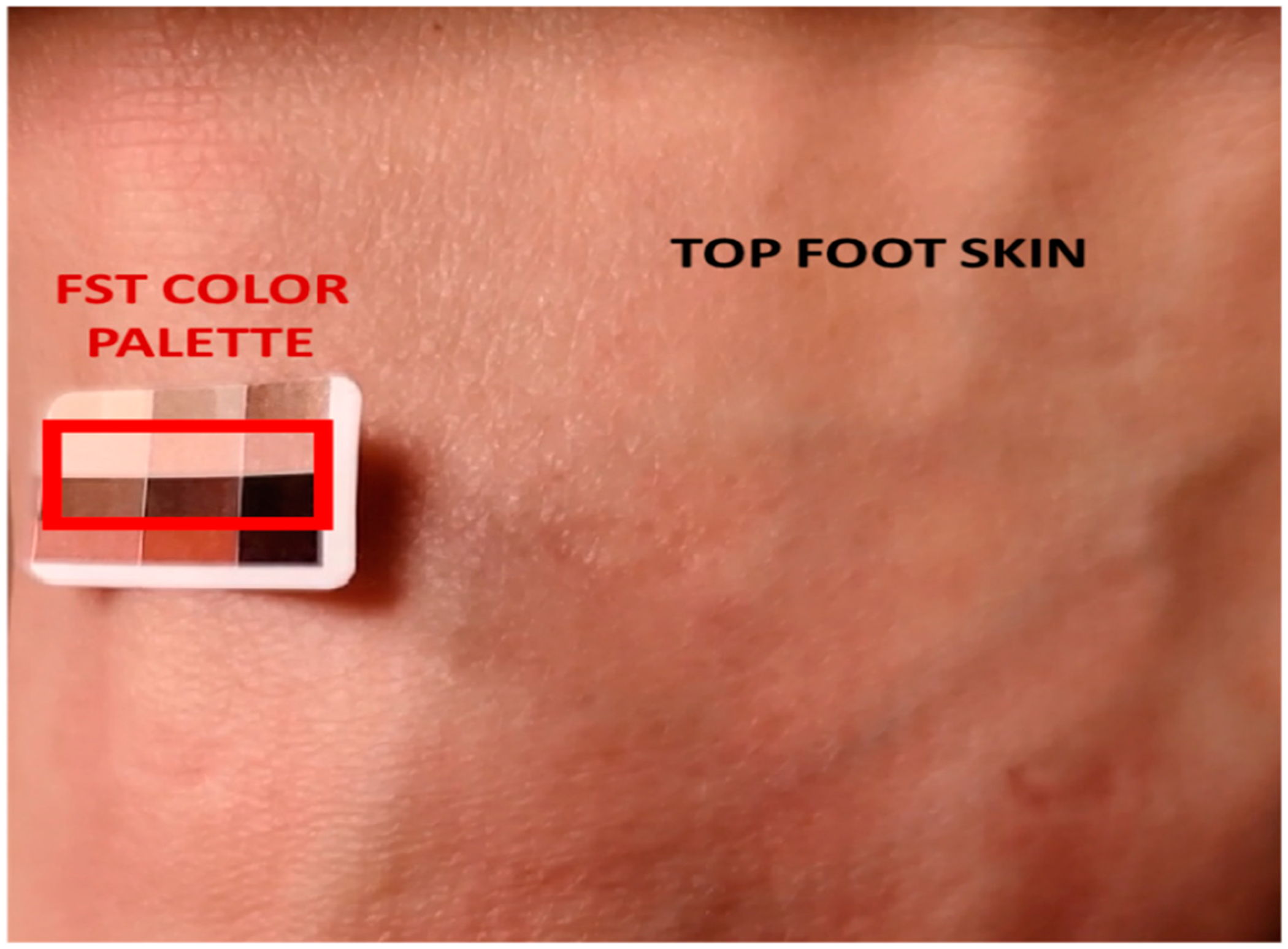
Sample of top-foot skin and FST scale palette imagery used in the comparative study of clustering models.

A specific subset from the collected dataset was meticulously selected for this comparative analysis. This subset comprised two key images: one representing the skin color (top-foot location) of the human subject, and the other featuring the FST palette scale used for the matching (Figure 5). These images were chosen to provide a focused and representative sample for evaluating the performance of various clustering models, thereby enabling a precise and targeted analysis within the broader dataset.

**Figure 6.**
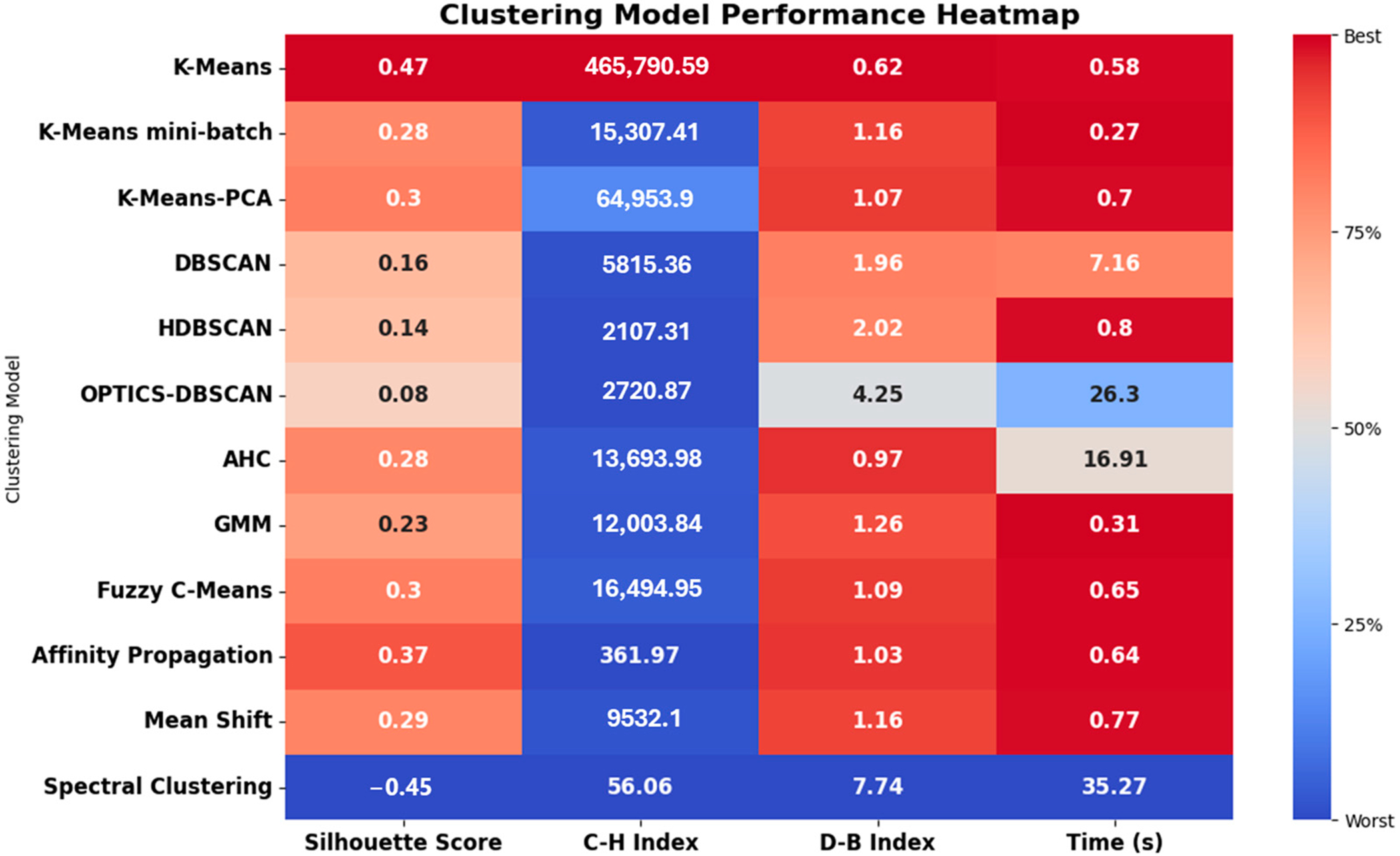
Comparative visualization of clustering model performances in AIDA system.

The comparative performance analysis of the clustering models considered here is depicted in Figure 6.

- K-means-type models: K-means demonstrated superior performance, with a higher silhouette score (0.47) and C-H Index (465,790) compared to K-means mini-batch and K-means-PCA, indicating better cluster quality and separation. However, K-means mini-batch had a shorter training time (0.27 s), suggesting greater computational efficiency, albeit at the cost of clustering quality. K-means-PCA, an extension of K-means with dimensionality reduction, showed a moderate silhouette score and C-H Index (0.28 and 15,307, respectively), suggesting decent clustering but not as effective as standard K-means. The incorporation of PCA appeared to slightly increase the training time (0.7 s) compared to basic K-means.
- DBSCAN-type models: DBSCAN and HDBSCAN, both density-based models, exhibited lower scores across all performance metrics compared to K-means. Their lower silhouette scores (0.16) indicate less distinct clustering, which might be due to the complex nature of skin color data not conforming well to density-based clustering. OPTICS-DBSCAN performed poorly in comparison to other models, with the lowest silhouette score and the highest D-B Index (0.08 and 4.25, respectively), indicating poor clustering quality and separation. Its significantly longer training time (26.3 s) also makes it less desirable for real-time application.
- Agglomerative hierarchical clustering (AHC): AHC showed moderate performance (silhouette score of 0.28) but required significantly more time for training (16.91 s), making it less suitable for scenarios where time is crucial.
- Gaussian mixture models (GMMs): GMMs presented a balance between cluster quality (with a silhouette score of 0.23) and training time (0.31 s) but did not excel in any metric.
- Fuzzy C-means: Fuzzy C-means, allowing for overlapping clusters, showed reasonable performance (with a silhouette score of 0.3), suggesting its potential applicability in situations where skin colors do not distinctly belong to separate categories.
- Affinity propagation and mean shift: Both of these models demonstrated moderate to high silhouette scores (with a silhouette score of 0.37 for affinity propagation and 0.29 for mean shift) but were not as effective as K-means in overall clustering performance.
- Spectral clustering: Spectral clustering was found to be the least suitable for this application, evidenced by its negative silhouette score (−0.45) and the longest training time (35.27 s), indicating poor clustering effectiveness and computational inefficiency.

Based on these results, standard K-means emerged as the most effective model for skin color classification in the AIDA system, offering a balance between clustering quality and computational efficiency. While other models like K-means mini-batch and Fuzzy C-means showed potential in specific contexts, their overall performance was outshined by K-means. The comparative analysis underscores the importance of selecting a model that not only provides accurate clustering but also aligns with the practical requirements of speed and efficiency in a clinical setting.

### 3.4. Comparison with Supervised Learning Model

A critical comparative analysis was conducted between the best unsupervised clustering model (i.e., K-means model) and a supervised learning model, specifically the convolutional neural network (CNN). This comparison encompassed the entire dataset of 48 human subjects, providing a comprehensive understanding of the performance dichotomy between these two approaches in the context of skin color classification.

The primary criterion for comparison was the accuracy of classification against the ground-truth data (obtained using the colorimetry-based tool Delfin Skin ColorCatch). The effectiveness of both the unsupervised clustering model and the CNN was measured by how closely their classification of the dataset aligned with this predefined ground truth.

The K-means clustering models was first applied to the dataset. The K-means model classified the skin colors of the 48 subjects without prior labeling, relying solely on the inherent patterns and characteristics identified within the data. In parallel, a CNN model was trained and then used to classify the same dataset. The CNN was pre-trained with labeled data (obtained using Delfin Skin ColorCatch) to recognize and classify skin colors. Details of the architecture, data preparation, training, and evaluation of CNN model are provided in the next section.

Both methodologies were then evaluated on their accuracy, with their results compared to the ground-truth data. The evaluation metric was the classification accuracy, which was calculated as the percentage of correctly classified instances out of the total instances. Other classification metrics, such as precision, recall, and F1-score, were also considered to provide a complete view of the models’ performance.

The detailed description of the data preprocessing, augmentation techniques, CNN architecture, and training process has been explained and expanded in Appendix B. This includes information on the Bayesian optimization process used to fine-tune the CNN’s hyperparameters (such as kernel size, dropout rates, and learning rate), ensuring optimal performance.

#### Evaluation of K-Means AIDA Versus CNN Performance in Skin Color Classification

**Figure 7.**
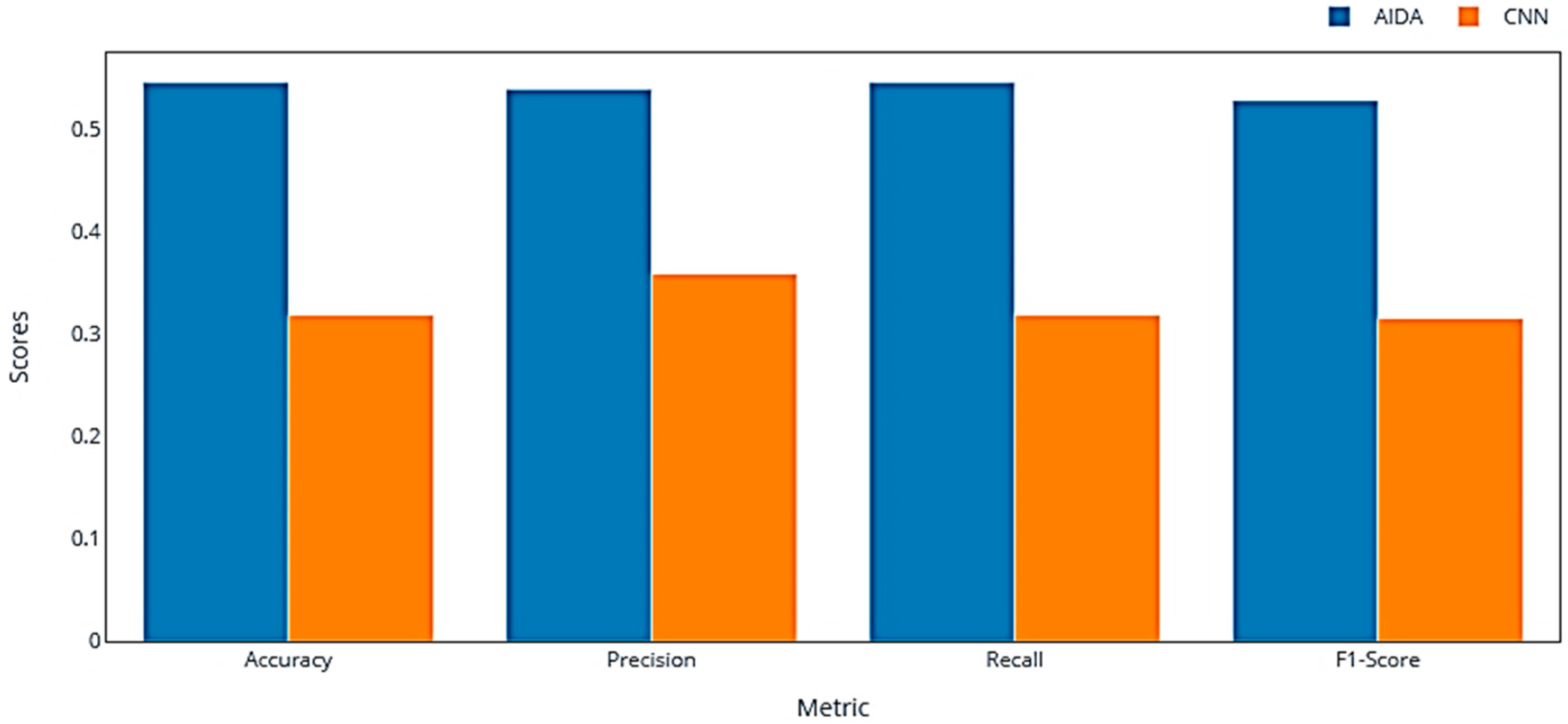
Bar chart for the evaluation of K-means AIDA vs. CNN performances in skin color classification.

The evaluation analysis conducted within the context of the AIDA project, comparing the performance of the K-means AIDA and CNN model, yielded notable results. The assessment focused on the accuracy, precision, recall, and F1-score of both models in classifying skin colors against the ground-truth data (Figure 7).

The AIDA system exhibited a notable performance in skin color classification, with an accuracy of 0.56, indicating that it correctly identified more than half of the skin colors when compared to the ground-truth data. This level of accuracy suggests a notable capability of the model in accurately predicting skin color categories. The precision of AIDA, which assesses the proportion of true positives among all positive predictions, was recorded at 0.54. This precision score implies a reasonably good tendency of the model to correctly classify skin colors when it predicts a specific category. Furthermore, the recall for AIDA was measured at 0.54, signifying that the model correctly identified approximately 54% of all relevant instances as per the ground truth. This recall score underscores the model’s effectiveness in detecting true positives. The F1-score, a critical metric that combines precision and recall, stood at 0.53 for AIDA. While this score highlights a balance between precision and recall, it also indicates areas where the model’s overall accuracy and reliability could be enhanced. These results reflect the proficiency of the AIDA system in classifying skin colors, with its performance metrics demonstrating a substantial degree of accuracy, precision, and recall in line with the objectives of the study.

The CNN model demonstrated an accuracy of 0.32, suggesting that only 32% of the classifications matched the ground-truth data. This lower accuracy indicates significant challenges in the model’s ability to correctly classify skin colors. With a precision score of 0.36, the CNN showed a lower likelihood of correct positive predictions compared to AIDA. This lower precision points towards a higher rate of false positives in the CNN’s classifications. The recall for the CNN was 0.32, which means it correctly identified 32% of all relevant instances. This lower recall score indicates a reduced sensitivity in detecting true positives. The F1-score for the CNN stood at 0.31, significantly lower than AIDA’s score. This lower F1-score reflects a suboptimal balance between precision and recall, emphasizing the model’s limitations in both aspects.

The comparative evaluation revealed that the K-means clustering algorithm used in AIDA outperformed the CNN model across all metrics. While AIDA demonstrated moderate effectiveness in classifying skin colors, the CNN model exhibited notable challenges, evident in its lower accuracy, precision, recall, and F1-score. These results underscore the potential of the AIDA approach in effectively handling complex tasks like skin color classification, especially when compared to traditional supervised approaches like CNNs.

### 3.5. Performance Analysis of AIDA Versus CNN with Tolerance

**Figure 8.**
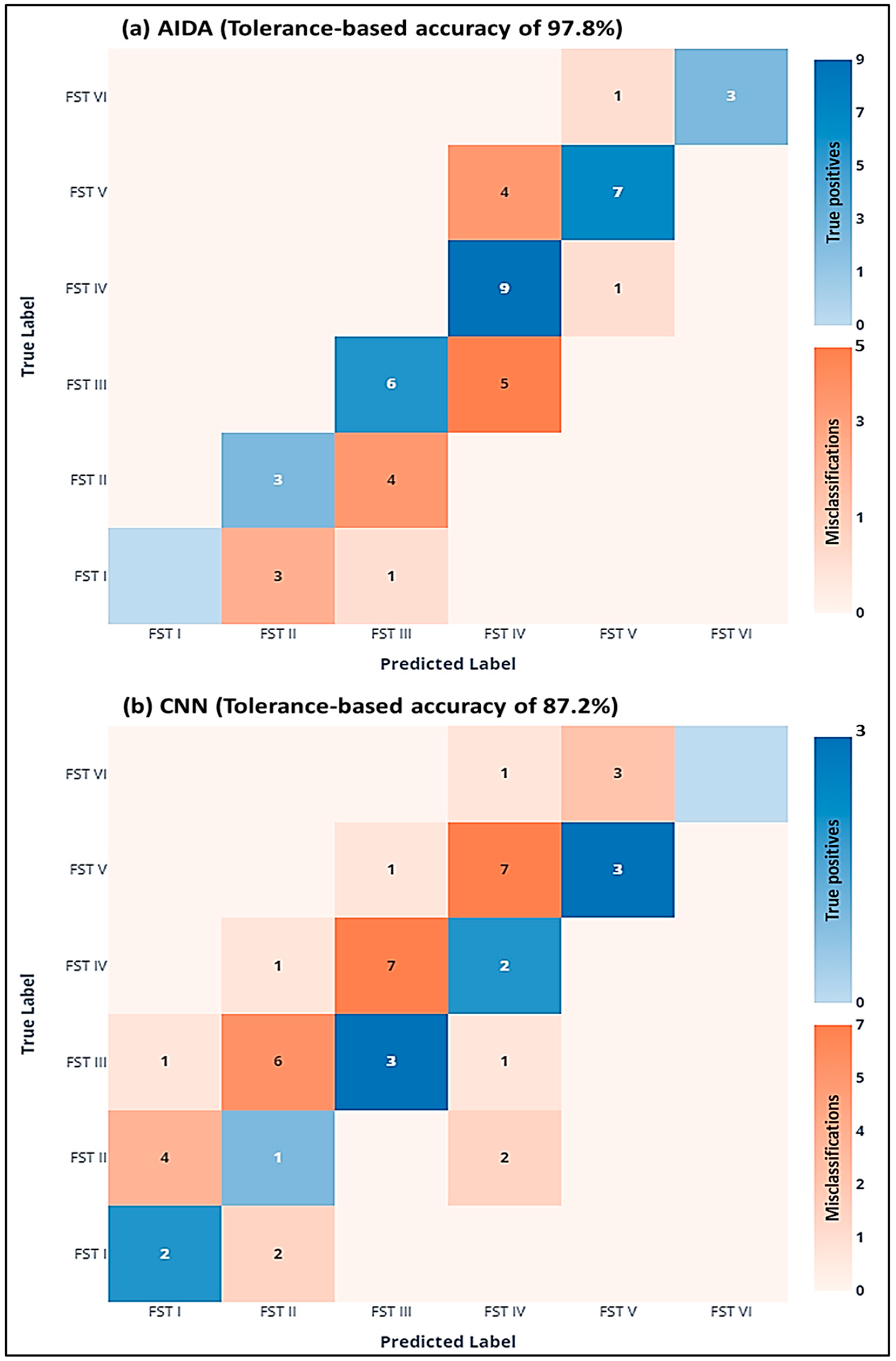
Confusion matrices for the comparative analysis of (**a**) AIDA and (**b**) CNN predicted outcomes vs. ground-truth FST classes.

Adopting a practical approach with a ±1 tolerance level for predictions revealed another significant distinction between the two models (Figure 8). AIDA achieved a remarkable tolerance-based accuracy of 97%, showcasing its consistency and reliability within a clinically acceptable margin of error. In contrast, the CNN model attained an 87% accuracy under the same criteria. This difference underscores AIDA’s enhanced capability to match the ground-truth values more closely.

The visualizations further supported this finding, with AIDA’s predictions displaying a tighter concentration around the perfect prediction line, especially within the ±1 deviation band. This contrasted with the CNN’s broader distribution, as seen in both the scatter plots and the histograms. The performance with a ±1 tolerance highlights AIDA’s robustness in predicting FST colors, affirming its superiority over the CNN model. AIDA not only excels in exact match accuracy but also demonstrates greater adaptability and precision in a clinical context, where a margin of tolerance is often necessary. The comprehensive evaluation reveals AIDA’s potential as a more effective tool for dermatological assessments and research into skin color classification. Its higher tolerance-based accuracy reflects AIDA’s advanced predictive capabilities, making it a preferred choice for applications requiring nuanced skin color analysis.

### 3.6. Spatial Mapping of Skin Regions Using AIDA

**Figure 9.**
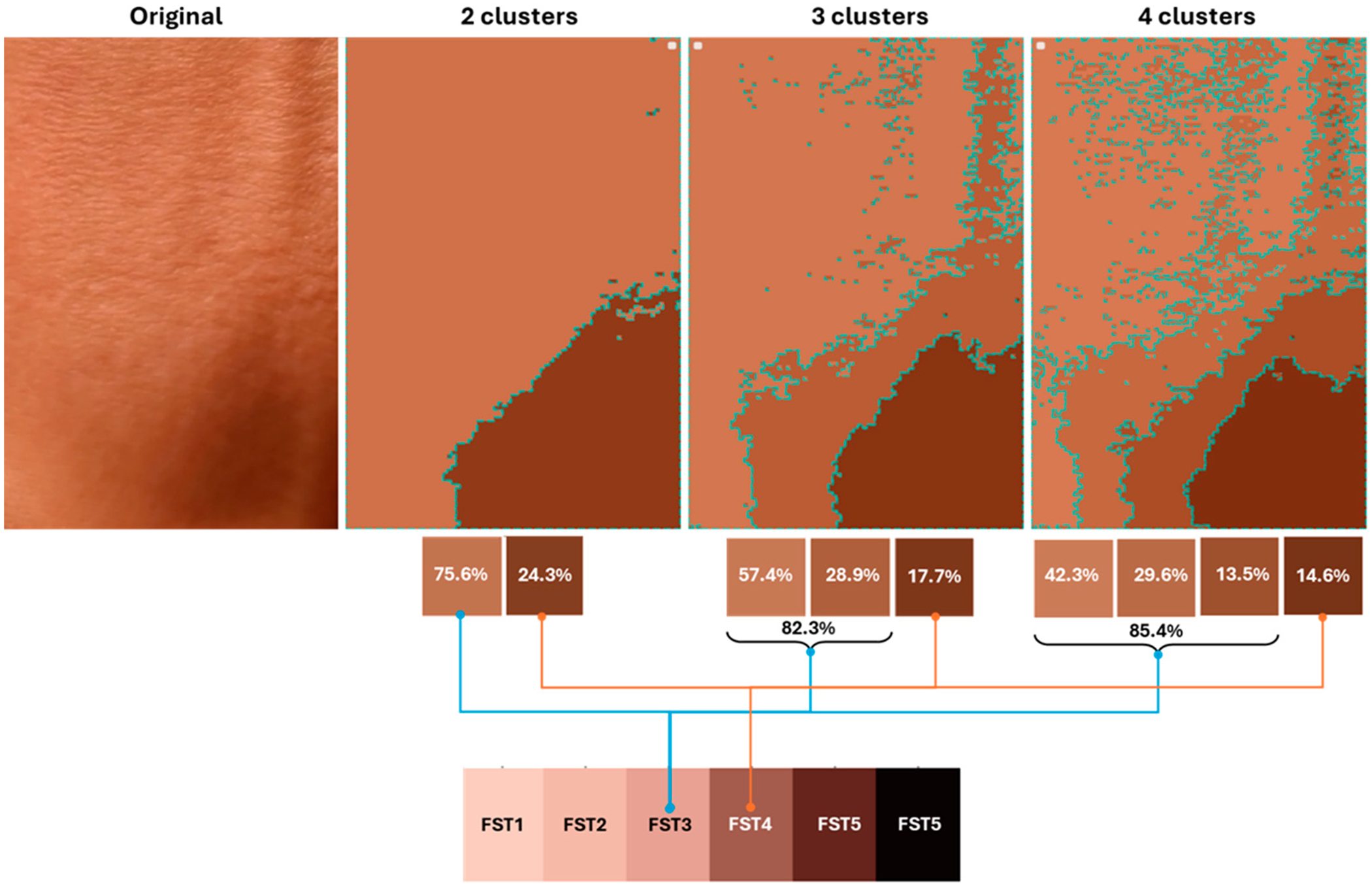
Skin regions using AIDA algorithm with two, three, and four cluster segments, matched to FST categories.

The AIDA clustering algorithm can also be applied to segment the skin image into distinct clusters based on color similarity. Each cluster is then matched with the closest FST category using the earlier principle of the Euclidean distance matching technique. The resulting spatial mapping provides a detailed visualization of the skin regions and their corresponding FST classifications. Figure 9 shows a single skin image (from one subject) processed using the AIDA algorithm, with two, three, and four cluster segments. Each segment was analyzed and matched with an FST category, demonstrating the ability to differentiate between subtle variations in skin tone. The AIDA system minimizes the effects of lighting and shadows by preprocessing images in the LAB color space, which is less sensitive to lighting variability than RGB. Additionally, clustering is based on Euclidean distances in color space, enabling the system to effectively differentiate skin tones from lighting artifacts. From the figure, it is evident that the predominant segment, which matches FST3, increases in size as the clusters progress from two to three to four (75.6, 82.3, 85.4%). Conversely, the segment matching FST4 decreases in size (24.3, 17.7, 14.6%). This dynamic adjustment contributes to reducing the uncertainty in skin color classification, enhancing the precision of the analysis.

## 4. Discussion

Through evaluating a variety of unsupervised learning models, notable differences in performance have been highlighted, particularly underscoring the robustness of the K-means clustering model. This superior performance is attributed to several key factors. The perceptual uniformity of the LAB color space [38] is conducive to the Euclidean distance measure utilized by K-means, ensuring that the visual importance of color changes is consistently maintained. Furthermore, the distribution of skin colors, which often naturally form compact clusters, aligns well with the spherical clustering tendency of K-means [39]. The algorithm’s reliance on centroids for defining clusters [25] is particularly advantageous for representing typical skin tones, a feature that holds significant value in dermatological diagnostics. K-means’ computational efficiency and resilience to lighting variations in the LAB color space [40], which are critical in dermatological analysis, also stand out. However, the efficiency–quality trade-off with K-means mini-batch and the minimal performance impact of PCA integration require further exploration [41,42]. Challenges such as the lower performance of density-based models [27] and the long training times of models like Spectral Clustering [43,44] also stress the need for careful model selection based on specific dataset characteristics. The promising performance of fuzzy C-means [45] suggests potential for applications requiring nuanced skin color analysis, reflecting the complexity of human skin tones. In the context of the AIDA project’s evaluative research, another comparative analysis was conducted to assess the efficacy of the AIDA clustering-matching algorithm against that of a CNN model in the domain of skin color classification. This examination was meticulously structured around key performance indicators including accuracy, precision, recall, and the F1-score, with the objective of delineating the comparative merits of each model in aligning classifications with ground-truth data. The results derived from this comparative study underscored a notable proficiency of the AIDA system, employing the K-means clustering algorithm combined with a matching technique, in the classification of skin colors. An accuracy metric of 0.56 was recorded for AIDA, indicating a successful classification of more than half of the skin color samples in concordance with the ground truth. This level of accuracy signifies a commendable predictive capability inherent within the AIDA model. Precision for AIDA was documented at 0.54, revealing a reasonable efficacy of the model in generating true positive classifications amidst its predictions. Furthermore, a recall rate of 0.54 was observed, suggesting that the AIDA model was capable of correctly identifying a significant proportion of true positive instances in accordance with the ground truth. The F1-score, a harmonic mean of precision and recall, was determined to be 0.53 for AIDA, indicative of a balanced trade-off between the precision and recall metrics, albeit highlighting potential avenues for enhancing the model’s classification performance.

The CNN model demonstrated significantly lower efficacy in skin color classification, with an accuracy of only 0.32, indicating that its classifications aligned with the ground truth in just 32% of instances. This low accuracy highlights major challenges in the model’s performance. It reported a precision of 0.36 and a recall of 0.32, both reflecting its limited ability to predict and identify true positives accurately. The CNN’s F1-score, at 0.31, also substantially trailed behind that of AIDA, underscoring its difficulty in balancing precision and recall effectively. A performance analysis, incorporating a ±1 tolerance level for the prediction of skin colors, revealed a pronounced distinction between the AIDA and CNN models. A tolerance-based accuracy of 97.8% was achieved by AIDA, illustrating its substantial consistency and reliability within a margin of error deemed clinically acceptable [46,47]. In comparison, the CNN model exhibited an 87% accuracy under identical conditions, highlighting AIDA’s superior precision in closely matching ground-truth values. AIDA’s predictions demonstrated a notable concentration around the ideal prediction line, particularly within the ±1 deviation range. This was in stark contrast to the CNN’s predictions, which were characterized by a broader dispersion, as evidenced in both scatter plots and histograms. The enhanced performance observed with a ±1 tolerance underscores AIDA’s robustness in predicting Fitzpatrick Skin Type (FST) colors, affirming its dominance over the CNN model. The analysis elucidates how AIDA not only excels in achieving exact match accuracy but also in achieving superior adaptability and precision within a clinical setting, where tolerances are often indispensable. This distinction accentuates the potential of AIDA to significantly advance the field of dermatological diagnostics, offering a more nuanced and accurate approach to skin color classification that accommodates the inherent variability and complexity of human skin tones. The AIDA clustering algorithm effectively segments skin images into distinct clusters based on color similarity, matching each with the closest Fitzpatrick Skin Type (FST) category. Figure 9 illustrates how the algorithm processes a single skin image, increasing the size of the predominant class segment (FST3 in the example) as clusters progress from two to four while the other segment (FST4) decreases. This dynamic adjustment reduces uncertainty in skin color classification and enhances analysis precision.

The deployment of AIDA is user-friendly due to its foundation in unsupervised learning, which minimizes the need for manual intervention and reliance on labeled datasets. This characteristic simplifies the integration process into diverse dermatological workflows. Future development plans for AIDA include the creation of an intuitive dashboard to facilitate the visualization of clustering results and skin tone classifications. Additionally, AIDA is being tailored for compatibility with the SPOT device [7,34]. This integration is expected to offer a compact and accessible solution, further enhancing the practicality of the system in real-world applications.

Additionally, while this study was conducted in a controlled environment to establish a baseline for the performance of AIDA, addressing its adaptability to real-world conditions remains a priority for future work. Practical deployment scenarios often involve variations in lighting and imaging conditions, which could impact the system’s robustness. To simulate these practical settings, we plan to evaluate AIDA under a range of lighting conditions, including diverse color temperatures and ambient light intensities. Furthermore, specific guidelines will be developed for camera specifications, such as resolution, sensor quality, and dynamic range, to standardize image capture across different devices. These efforts aim to enhance AIDA’s reliability and adaptability, ensuring consistent performance in diverse clinical and field applications.

## 5. Conclusions

The AI Dermatochroma Analytica (AIDA) approach represents a significant leap forward in the field of dermatological research, particularly in the classification and analysis of human skin colors. Our study has successfully demonstrated the ability of AIDA to overcome the limitations of other skin color classification methods, such as subjective visual assessments and actual image processing systems. By employing unsupervised learning algorithms, AIDA has effectively transcended the constraints of conventional approaches, offering a more nuanced and accurate understanding of the complex spectrum of human skin colors.

A key finding of our research is the superior performance of AIDA’s K-means clustering model compared to a supervised convolutional neural network (CNN). AIDA’s approach resulted in double the performance rate of CNN in skin color classification, highlighting its efficiency and effectiveness in handling the diversity and complexity inherent in human skin. Furthermore, the inclusion of a tolerance-based evaluation strategy, reflecting realistic clinical scenarios, resulted in an impressive 97% accuracy (versus 87% with CNN), reaffirming AIDA’s robustness and reliability in predicting skin colors within a clinically acceptable range. Additionally, the AIDA clustering algorithm effectively segments skin images into distinct clusters based on color similarity, matched with the closest FST category. The spatial mapping from these clusters provides a detailed visualization of skin regions, reducing uncertainty in skin color classification and enhancing precision.

The flexibility of AIDA to adapt to various skin color scales, coupled with its integration of the FST, underscores its potential as a versatile tool in dermatology. This adaptability ensures that AIDA remains relevant across diverse geographical and ethnic landscapes, as well as in the light of emerging dermatological research. The efficacy of AIDA in classifying skin colors is notably sensitive to the quality of lighting and the camera used for capturing images. Consistent and appropriate lighting conditions are equally crucial, as variations in lighting can significantly impact the perception and representation of skin colors. Maintaining uniform lighting during the image capture process is essential to minimize any distortions or inconsistencies in the skin color data. Adherence to these standards will greatly enhance the precision and reliability of AIDA’s skin color classification, thereby optimizing its performance in dermatological applications.

In conclusion, the AIDA system marks a significant advance in dermatological technology. Its innovative approach, combining advanced machine learning techniques with dermatological expertise, sets a new standard for skin color analysis. The results of this study pave the way for more accurate, efficient, and personalized dermatological care. Moreover, the AIDA system holds great potential for applications in the cosmetics industry, enabling personalized product recommendations based on precise skin tone analysis, improving shade matching, and enhancing user satisfaction. The future development of AIDA promises significant advancements in dermatology. Key areas for growth include expanding the dataset to encompass a wider range of skin colors, particularly from under-represented demographics. Furthermore, adapting AIDA for the identification and assessment of various skin conditions, such as pigmentation disorders, physiological assessment of wounds in diabetic feet of members of any racial/ethnic group, or early detection of skin cancers, can broaden its clinical utility.

## Figures and Tables

**Table 1.** Clustering models evaluated in AIDA.

Clustering Model	Description	Refs.
K-means models	K-means and its variants divide data into clusters by iteratively minimizing the sum of squared distances between data points and their assigned cluster centroids. Variants like K-means-PCA reduce dimensionality, while K-means mini-batch optimizes for efficiency.	[25,26]
Density-based models	Models like DBSCAN, HDBSCAN, and OPTICS identify clusters by grouping data points with sufficient density, effectively detecting arbitrarily shaped clusters and outliers in sparse data.	[27]
Hierarchical methods	Agglomerative hierarchical clustering (AHC) builds a tree-like structure by iteratively merging or splitting clusters based on their similarity, enabling the exploration of data at multiple granularity levels.	[28]
Probabilistic techniques	Gaussian mixture models (GMM) use a probabilistic approach to model data as a mixture of multiple Gaussian distributions, assigning probabilities for data point membership in overlapping clusters.	[29]
Fuzzy logic approaches	Fuzzy C-means assigns data points to multiple clusters with varying degrees of membership, reflecting the inherent ambiguity in boundaries between certain skin color categories.	[30]
Other clustering methods	Models such as affinity propagation identify exemplars for clusters by passing messages between data points, mean shift locates cluster centers by maximizing density, and spectral clustering partitions data using eigenvalues of a similarity matrix.	[31–33]

**Table 2.** Study design and data collection.

Aspect	Details
Study location and approval	Conducted at Florida International University (FIU) under IRB-13-0092, focusing on capturing white light data using the smartphone oxygenation tool (SPOT) device [7,34] for skin color classification [23].
Subjects	A total of 48 control subjects across FST I to VI were recruited. Subjects were seated or supine with feet exposed for imaging.
Imaging setup	A reference sticker with six FST colors was placed within the imaging field of view. A black curtain provided a consistent background, isolating the foot.
Imaging process	Images were captured at 7 foot locations under three lighting conditions. The top-foot location under a controlled lighting condition (4100 K) was used for the proof-of-concept.
Ground-truth comparisons	Skin color classifications by a researcher, a clinician, and a commercial colorimetry-based tool (Delfin Skin ColorCatch) were compared for consistency and variability. Researcher and clinician classifications were completed by visual comparison on the FST scale and showed significant subjectivity, with variability between researcher and clinician classifications. The commercial device provided predominantly consistent results, unaffected by external lighting, and was chosen as the ground truth (Appendix A Figure A1).

**Table 3.** Study design and data collection.

Metric	Definition	Dermatological Relevance
Silhouette score [35]	Measures the degree of similarity of an object within its own cluster compared to others. Values range from −1 to +1, with higher values indicating better cohesion and separation.	Ensures each skin tone cluster aligns distinctly with an FST category, aiding in the evaluation of cohesion and separation.
Calinski–Harabasz (C-H) Index [36]	Known as the variance ratio criterion, it measures dispersion between and within clusters. Higher scores indicate more distinct clustering.	Quantifies the distinctiveness of skin tone clusters, ensuring well-defined boundaries between FST categories.
Davies–Bouldin (D-B) Index [37]	A ratio of within-cluster to between-cluster distances, indicating compactness and separation. Lower values suggest better clustering.	Helps assess partitioning effectiveness, reducing overlap between FST categories for precise classifications.
Training Time	Elapsed time required for the system to train the model, recorded in seconds to evaluate computational efficiency.	Evaluates computational efficiency, ensuring suitability for real-time clinical applications.

Details of the performance metrics utilized in this study are outlined in Appendix A.

**Figure A1.**
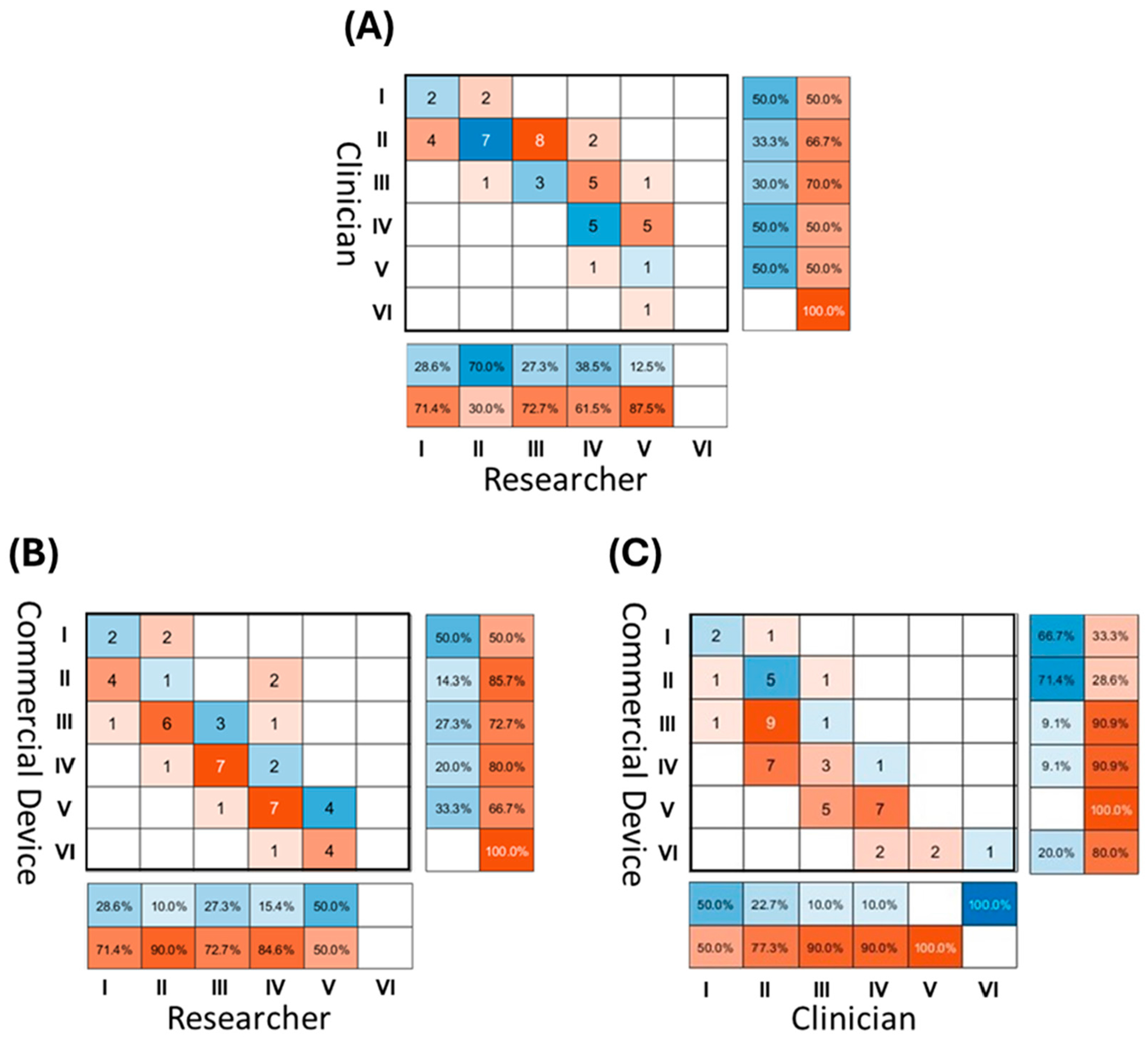
Confusion matrix comparison of three ground-truth methods that could be used for testing the unsupervised convolutional network model from 48 images. Red coloration signifies a mismatch, while blue is a match for FST class. (**A**) Visual classification by a clinician versus visual classification by researcher, both using an FST sticker within image. (**B**) Commercial device FST classification using individual topological angle (ITA) measurements versus visual classification by researcher using an FST sticker within image. (**C**) Commercial device FST classification using individual topological angle (ITA) measurements versus visual classification by clinician using an FST sticker within image.

**Figure A2.**
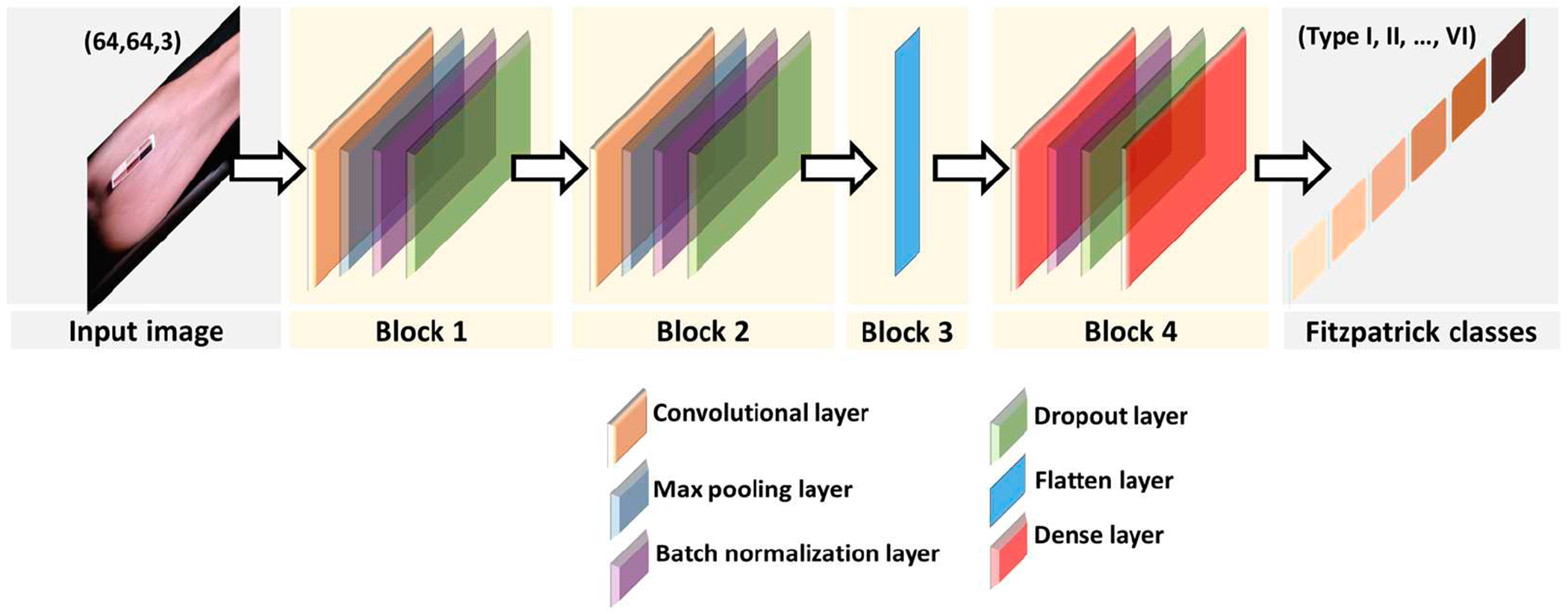
Architecture of convolutional neural network (CNN) model used in the comparative analysis.

## Data Availability

The data presented in this study maybe available upon request from the corresponding author. The data are not publicly available due to restrictions imposed by the funding party, which limit data sharing to protect proprietary information and intellectual property. Access to the data will be granted upon reasonable request, subject to approval from the funding party.

## Appendix A

**Table A1.** Specific libraries imported and their primary uses.

Task	Tool Description
Image processing (OpenCV)	OpenCV (Open-Source Computer Vision Library) [48] was integral for image processing and computer vision tasks. It facilitated crucial operations such as reading, resizing, and transforming images, as well as converting them between color spaces.
Data processing (NumPy/Pandas)	The incorporation of the NumPy and Pandas libraries [49] was critical in managing and processing data for machine learning applications. NumPy, renowned for its capabilities in numerical computing, was primarily utilized for its efficient array (vector, matrix) operations.
Machine learning (Scikit-Learn)	The integration of Scikit-Learn and Scikit-Image libraries played a crucial role in both machine learning and image processing aspects. Scikit-Learn [50], a prominent machine learning library, was utilized to implement the clustering algorithm.
Plotting and visualization (Seaborn/Matplotlib)	Seaborn and Matplotlib libraries were utilized to facilitate advanced data visualization. Seaborn [51] offered a high-level interface for creating aesthetically pleasing and informative statistical graphics.

### Appendix A.1. Details About Performance Metrics

- Silhouette score is calculated using the following formula [35]:
  (A1)
  $$S(i)=\frac{b(i)-a(i)}{\max\{a(i),b(i)\}}$$
  where *a*(*i*) is the average distance from the *i*th data point to the other points in the same cluster, and *b*(*i*) is the smallest average distance from the *i*th data point to points in a different cluster, minimized over all clusters.
  This score, ranging from −1 to +1, is employed as a metric to determine the degree of similarity an object holds within its own cluster in comparison to other clusters. Higher values in the silhouette score indicate a strong match to the respective cluster and a poor match to neighboring clusters. In the context of dermatology, the silhouette score is crucial for evaluating the cohesion and separation of skin tone clusters, ensuring that each identified skin tone distinctly aligns with a specific Fitzpatrick Skin Type (FST) category.
- Calinski–Harabasz (C-H) Index is defined by the following formula [38]:
  (A2)
  $$C-H(k)=\frac{B(k)/(k-1)}{W(k)/(n-k)}$$
  where *B*(*k*) is the between-group dispersion matrix, *W*(*k*) is the within-cluster dispersion matrix for *k* clusters, and *n* is the number of data points.
  Also known as the variance ratio criterion, this index is a measure of the dispersion between and within clusters. Elevated scores on this index suggest more distinct clustering. For dermatological applications, the C-H Index quantifies the distinctiveness of skin tone clusters, ensuring that the boundaries between Fitzpatrick Skin Types are well-defined.
- Davies–Bouldin (D-B) Index is determined using [37]:
  (A3)
  $$D-B=\frac{1}{n}\sum_{i=1}^{n}\max_{i\neq j}\left(\frac{\delta \left(c_{i},c_{j}\right)}{\text{Δ}\left(c_{i}\right)+\text{Δ}\left(c_{j}\right)}\right)$$
  Here, *$\delta \left(c_{i},c_{j}\right)$* represents the distance between centroids of clusters *i* and *j*, and $\text{Δ}\left(c_{i}\right)$ is the average distance of all points in cluster *i* to the centroid *c_i_*.
  This index, a function of the ratio of within-cluster to between-cluster distances, is indicative of the compactness and separation of clusters. Lower values in the index are indicative of better clustering. From a dermatological perspective, the D-B Index helps assess how well the clustering algorithm partitions skin tone data into compact and clearly separated categories. This is essential for reducing overlap between Fitzpatrick Skin Types, ensuring precise and reproducible classifications in dermatological practice.
- Training time: This metric is measured as the elapsed time required for the system to train the model. It is recorded in seconds. The computational efficiency of AIDA was evaluated through the measurement of the time elapsed, ensuring its suitability for real-time applications in clinical environments.

## Appendix B

### Appendix B.1. Data Preprocessing and Augmentation for CNN

A comprehensive approach was adopted for data preprocessing and augmentation for the CNN used in skin color classification. This process involved several image manipulation techniques to enhance the diversity and quality of the dataset, ensuring robust training and evaluation of the CNN model.

- Data preprocessing techniques: Two key image preprocessing steps were employed to enhance skin color analysis. First, a function cropped out white borders by converting images to grayscale, thresholding for white regions, and then cropping to the largest contour’s bounding box. Second, white frames were removed using advanced morphological operations and contour detection, ensuring only relevant skin color information was retained. These steps were pivotal in focusing on essential skin areas and eliminating irrelevant content.
- Data augmentation techniques: To increase the dataset’s variability and simulate different real-world conditions, several augmentation techniques were applied. These included rotating images at random angles and random horizontal and vertical shifts for variability in color positioning. Additionally, random zooming simulated varying camera-subject distances, while horizontal and vertical flipping diversified the dataset by mirroring skin presentations. A batch augmentation process was developed to systematically apply these techniques, generating multiple augmented versions of each image. This expanded the dataset significantly, with the augmented images saved for use in model training and validation.
- Data oversampling technique: To tackle the issue of uneven FST distribution in the dataset, we used the oversampling technique to ensure a fair representation for each FST. This expanded the dataset to include 1000 subject images for every FST. This approach addressed the class imbalance in the training data. The combination of these preprocessing and augmentation techniques resulted in a richly varied and high-quality dataset, crucial for the effective training of the CNN model.

### Appendix B.2. Architecture and Training of CNN Model

After data augmentation, each image was resized to 64 × 64 pixels to standardize the input size and ensure faster training. Images were also normalized by dividing pixel values by 255, converting them into a range of 0 to 1. Skin colors were labeled based on the ITA measurements (provided by Delfin Skin ColorCatch) that were correlated to the FST class, providing an objective ground truth for training the CNN.

- Model architecture and hyperparameter tuning (Figure A2):

The CNN model included multiple convolutional layers with MaxPooling and Batch-Normalization. These layers are instrumental in extracting features from the images. Dropout and L2 regularization were used to prevent overfitting, ensuring the model’s generalizability to new, unseen data. The model concluded with dense layers, including a final SoftMax layer for classification, which maps the extracted features to the respective skin color categories.

A Bayesian optimization approach was used to find the best hyperparameters for the CNN. This included optimizing the number of filters, kernel size, dropout rates, and regularization parameters in convolutional and dense layers.

- Training and evaluation:

Early stopping and reduce learning rate on plateau were used as callbacks to enhance the training process, prevent overfitting, and adjust the learning rate for optimal convergence.

The CNN was trained on the prepared dataset, with the validation set used to monitor the model’s performance and prevent overfitting. During the training phase, 24 subjects were initially utilized, while the remaining 24 were set aside for testing the model’s performance on untrained data, for comparison with AIDA (Table A2). Employing extensive data augmentation techniques, the 24 subject images were expanded to 6000, featuring 1000 images per FST category. In the training process, 70% of the available data are allocated for training the convolutional neural network (CNN). The remaining 30% are strategically divided between validation and test sets to assess the model’s performance and generalization capabilities. The CNN model is precisely defined with specific hyperparameters, and Bayesian optimization is employed to search for the optimal configuration. Subsequently, the CNN is trained on the augmented and balanced training set, incorporating early stopping and learning rate reduction for effective convergence. Overall, the training process involves feeding the augmented and balanced dataset into the CNN model.

- Performance evaluation:

After training, the model was evaluated on the test set to determine its accuracy and effectiveness in classifying skin colors.

**Table A2.** Datasets for CNN training and for comparison analysis of AIDA vs. CNN.

Fitzpatrick Scale (FST)	Index of Subject Samples Utilized for CNN Training/Testing	Index of Subject Samples Utilized for Comparison Analysis of CNN vs. AIDA
FST—1	17, 29	32, 35
FST—2	4, 5, 6, 34	36, 38, 39
FST—3	7, 8, 9, 10, 15	21, 22, 26, 30, 33, 37
FST—4	1, 2, 3, 12, 16, 18	19, 23, 27, 28, 41
FST—5	13, 14, 25, 31, 40	42, 43, 44, 45, 48
FST—6	11, 24	46, 47
